# Investigating rumination and eating time as proxies for identifying dairy cows with low methane-emitting potential

**DOI:** 10.3168/jdsc.2024-0611

**Published:** 2024-09-20

**Authors:** A. Castaneda, N. Indugu, K. Lenker, K. Narayan, S. Rassler, J. Bender, L. Baker, O. Purandare, D. Chai, T. Webb, X. Zhao, D. Pitta

**Affiliations:** 1 Department of Clinical Studies, New Bolton Center, School of Veterinary Medicine, University of Pennsylvania, Kennett Square, PA 19348; 2 Department of Animal Science, McGill University, Sainte-Anne-de-Bellevue, Quebec H9X 3V9, Canada

## Abstract

Identifying cows with low CH_4_-emitting potential can greatly contribute to CH_4_ abatement in dairy herds. However, this process has been cumbersome and labor-intensive. Ear tags and collar-based accelerometers measure rumination and chewing behaviors, potentially identifying novel phenotypes in cows. This study aimed to determine whether rumination and eating time are linked to enteric CH_4_ emissions and serve as proxies to identify CH_4_ yield phenotype in lactating dairy cows. We applied the dynamic time warping model to rumination and eating time datasets to classify cows differing in these phenotypes. We calculated the distances between cows differing in rumination and eating times and depicted them in a principal component analysis plot. From 49 cows in early to mid lactation, 10 low-rumination, and 10 high-rumination cows were selected to test the relationship between rumination and eating time with CH_4_ yield phenotype over 7 wk. Enteric CH_4_ emissions were measured using the GreenFeed System. The dynamic time warping model identified cows with distinct rumination and eating patterns. High-rumination cows had higher DMI and milk yield, and lower enteric CH_4_ emissions than low-rumination cows. High-rumination cows also had lower CH_4_ intensity and higher production efficiency than low-rumination cows. Overall, rumination and eating time can be suitable proxies for identifying CH_4_ yield phenotype in dairy cows. Further studies, including larger dairy herds, different dietary regimens, and stages of lactation in dairy cattle and other ruminant species to validate rumination and eating time as proxies for identifying CH_4_ yield phenotype, are required.

## Highlights

• Precision livestock farming tools can detect low CH_4_-emitting cows.
• Applying the DTW model to RT and ET datasets, cows can be classified as LR and HR.
• HR cows have higher ET, DMI, MY, and lower EME than LR cows.
• RT and ET are “fingerprints” that can identify cows with low EME.
• RT and ET can be used as proxies to identify cows with low CH_4_-emitting potential.

## Graphical Abstract

**Figure:**
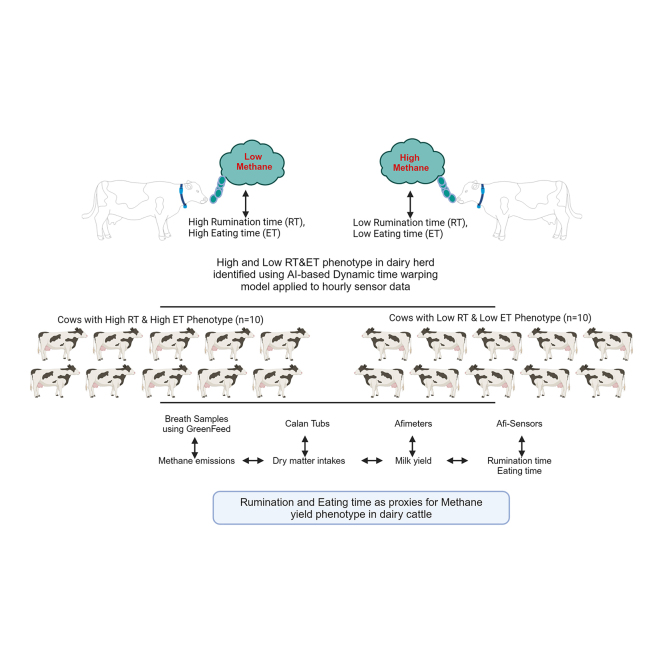
**Summary:** Combining precision livestock farming technologies, artificial intelligence-based algorithms, and behavioral responses allows for the identification of cows with low CH_4_-emitting potential. This process is possible by applying the dynamic time warping (DTW) model to rumination time (RT) and eating time (ET) datasets, allowing for the classification of cows into clusters of low rumination (LR) and high rumination (HR). We found that high-rumination cows had higher RT, dry matter intake (DMI), milk yield (MY), and lower enteric CH_4_ emissions (EME) than low-rumination cows. Furthermore, high-rumination cows had lower CH_4_ intensity and higher production efficiency than low-rumination cows. These data reveal that when precision livestock farming technologies and artificial intelligence-based algorithms were coupled to RT and ET datasets, it was possible to identify cows with low CH_4_-emitting potential.

A predominant factor driving global warming is the steady increase in the concentration of anthropogenic greenhouse gases, namely CO_2_, N_2_O, and CH_4_ in the atmosphere. As CH_4_ is more potent and short-lived than CO_2_ and N_2_O (USEPA, 2021, 2022), reducing its concentration has become a major priority. The sectors contributing to global CH_4_ emissions are oil and gas (24%), agriculture (22%), municipal solid waste (11%), coal mines (9%), wastewater (7%), and rice cultivation (7%) with other sources totaling 20% (IPCC, 2022; Bruns, 2023). In animal agriculture, most of CH_4_ emissions come from enteric fermentation (15%; Saunois et al., 2020; IPCC, 2022), mainly from cattle (Gerber et al., 2013). At the animal level, enteric CH_4_ emissions (**EME**) represent an “energy-inefficient” process. An adult cow can lose between 2% and 12% of its gross energy intake, which can vary depending on the nutritional content of the diet consumed (Johnson and Johnson, 1995). Thus, effective and novel strategies to mitigate the environmental and energy-inefficient costs of EME from cattle are required.

Among the strategies available to mitigate EME, identifying cows with low CH_4_ emitting potential is attracting scientists' attention (Almeida et al., 2021). Methane yield is moderately heritable (0.38) (Pickering et al., 2015; Manzanilla-Pech et al., 2021; Richardson et al., 2021), which can be incorporated into animal breeding programs to select cows with low CH_4_-emitting potential. However, screening cows to measure their EME is intensive and requires sophisticated equipment such as respiration chambers, GreenFeed System, or the sulfur hexafluoride tracer method. Moreover, given that prolonged sampling campaigns are required to collect representative data, measuring EME on large herds is impractical. Thus, implementing alternative methods to efficiently identify cows with low CH_4_-emitting potential is needed.

Precision livestock farming (**PLF**) technologies monitor cows' rumination, eating, and chewing patterns. Sensors such as the CowManager, Allflex, and the recently validated AFICollar (Afimilk, Kibbutz Afikim, Israel) provide information on physiological metrics such as rumination time (**RT**) and eating time (**ET**) from cows individually (Leso et al., 2021). Most rumination sensors provide values expressed in hours per cow per day for RT and ET, which can be linked to CH_4_ yield phenotype. However, such information is unsuitable for deriving meaningful patterns as the daily values can vary due to DMI, physiological status, diet composition, and the animal's health. Multiple studies have been conducted to determine the suitability of RT as a physiological response to identifying cows with low CH_4_-emitting potential (Mikuła et al., 2022); however, their results have been inconclusive.

Animal scientists are searching for proxies that are easy to measure, practical, and cheap for dairy producers to identify cows with low CH_4_-emitting potential. Several studies have investigated whether RT and CH_4_ yield phenotypes are associated so that RT can be used as a proxy for identifying cows with low CH_4_-emitting potential. Given that CH_4_ yield and RT are heritable and related to the mixing of ruminal contents and rumen physiology, it is reasonable to hypothesize that both traits are linked and that RT can serve as a marker for identifying cows with low CH_4_-emitting potential. Because rumination is heritable, each cow has its own “fingerprint.” These “fingerprints” are hidden in overwhelming RT datasets generated by PLF technologies. To handle these large and complex datasets, it is wise to recur to time series data mining tools. This resource provides algorithms aiming to find the best model and the best parameter value for a given dataset. Examples of models include the hidden Markov model (**HMM**), the longest common subsequence (**LCSS**), and the dynamic time warping (**DTW**) model (Li et al., 2020). The HMM assigns a statistical model to each trajectory to identify hidden patterns; however, this process is unsuitable due to its high time requirements. The LCSS model focuses on shape similarity, but similar to the HMM model, it has a high time cost. The DTW model is a time series alignment model that measures the similarity between sequences with varying speeds. It finds an optimal alignment by dynamically adjusting the time axis, making it effective for comparing sequences of different lengths or with temporal distortions. Although the DTW model is a nonlinear programming technique based on distance testing, it can calculate the similarity between 2 time series. As the efforts thus far in linking RT and ET with CH_4_ yield phenotype remain inconclusive, we also hypothesize that the DTW model may help find patterns in RT and ET datasets. Thus, we anticipate that the DTW model can find patterns in RT and ET datasets and link them to CH_4_ yield phenotype. If such a link exists, identifying cows with low CH_4_ emitting potential would be simple and RT and ET may be used as proxies to detect CH_4_ yield phenotype. Thus, this study aimed to investigate whether RT and ET are associated with CH_4_ yield phenotype by applying the DTW model to RT and ET datasets to identify lactating dairy cows with low CH_4_-emitting potential.

The protocol for animal handling and care was reviewed and approved by The University of Pennsylvania Institutional Animal Care and Use Committee under application number 80614. Our university herd was composed of Holstein lactating cows equipped with AFICollar (Afimilk, Kibbutz Afikim, Israel). We procured hourly RT and ET data from all cows collected from April 29, 2022, to June 27, 2022. We selected cows within 150 DIM to capture more stable performance during early to mid lactation. This criterion yielded 49 first-, second-, and third-lactating cows. We used the DTW model, which is available in the python package ‘dtaidistance' with a module ‘dtaidistance.dtw_ndim' (Shokoohi-Yekta et al., 2017) to calculate the distance between the pairs of the cows based on RT and ET. Using the generated DTW distance matrix, we computed agglomerative hierarchical clustering with the ‘agnes' function in the ‘cluster' package available in R (Mächler et al., 2017). The generated clusters were visualized in a principal component analysis (**PCA**) plot with the ‘fviz_cluster' function available in ‘factoextra' of R (Kassambara and Mundt, 2020). Last, we manually selected the cows to represent each cluster from the plot, resulting in 10 low rumination (LR cows; cluster 1) and 10 high rumination (HR cows; cluster 2). The cows that were on the extreme ends of the plot were selected and then blocked by parity, DIM, and milk yield (**MY**). The selected cows were fed a TMR once daily at 1000 h. A 10% refusal target was estimated based on the cow's daily feed intake. The TMR was prepared in a mixer, then deposited into a schooler, and finally distributed into individual tubs (American Calan Inc., Northwood, NH) using a motorized feeding vehicle (Data Ranger, American Calan Inc.). Each cow was assigned to a specific tub with doors controlled by radio frequency identification (RFID). Feeding into tubs allowed for calculating individual feed intakes and refusals. The feeding area was covered with rubber mats, which were manually scraped twice daily. The cows were milked twice daily at 0400 and 1500 h.

Before starting the experiment, the selected cows were assigned and trained to eat in their assigned tubs. Feed refusal samples were collected daily for DM and nutrient analysis from all tubs, after which the refusals were collected, weighed, and recorded using a motorized feeding vehicle. Feed samples were collected from the first, middle, and last tubs after each feeding event. The refusal and feed samples were stored at −20°C for future analysis. Enteric gas emissions (H_2_, CO_2_, and CH_4_) from individual cows were measured throughout the experiment using the GreenFeed System (C-Lock Inc., Rapid City, SD), which was calibrated according to the manufacturer's recommendations for accurate measurements. To collect breath samples, each cow was allowed to access the system for 5 min, followed by a 2-min waiting gap to avoid mixing the current breath sample with the next. The cows had free access to the GreenFeed System at all times.

The data were analyzed using PROC MIXED of the SAS statistical software (version 9.4 TS1M8 [9.4 M8], SAS Institute Inc.). The response variables were CH_4_, CO_2_, and H_2_ production, CH_4_ efficiency (CH_4_/DMI), CH_4_ intensity (CH_4_/MY), DMI, DM of refusals, MY, or feed efficiency. The fixed effect was cluster (LR and HR) and cow ID was the random effect. To assess the difference between the LR and HR cows on RT and ET data, we used generalized additive models (**GAM**) with integrated smoothness estimation. This model included RT and ET as response variables blocking by cluster (fixed effect) and cow ID (random effect). Hour and date were included as smoothing terms. Pearson correlation coefficients were computed to assess the association between EME with RT and ET at 2-h intervals. This analysis was performed while considering treatment groups collectively and separately for LR and HR cows. The correlation analyses were performed in R using the package ‘psych.'

We identified the clusters LR and HR in the selected cohort of cows (Figure 1). On average, LR cows ruminated 1,099 s h^−1^, ate 718 s h^−1^, produced 37.5 kg d^−1^ of milk, and were 44.5 DIM, whereas HR cows ruminated 1,296 s h^−1^, ate 852 s h^−1^, produced 44.2 kg d^−1^ of milk, and were 63.9 DIM. Animal performance variables were the milk annual average (44.2 vs. 37.5 kg d^−1^), milk total average (46.6 vs. 30.7 kg d^−1^), RT (554.9 vs. 467.8 min d^−1^), and ET (372.5 vs. 298.4 min d^−1^), which were all higher in HR cows relative to LR cows. Both clusters were further investigated for EME, DMI, MY, feed and production efficiency, RT, and ET for 7 wk. During the first 2 wk, the cows were trained to eat in their tubs. During wk 3 and 4, the cows were trained to visit the GreenFeed System to measure their EME. The experimental period occurred during wk 5 through 7 where EME and hourly physiological data for RT and ET were procured.

**Figure 1:**
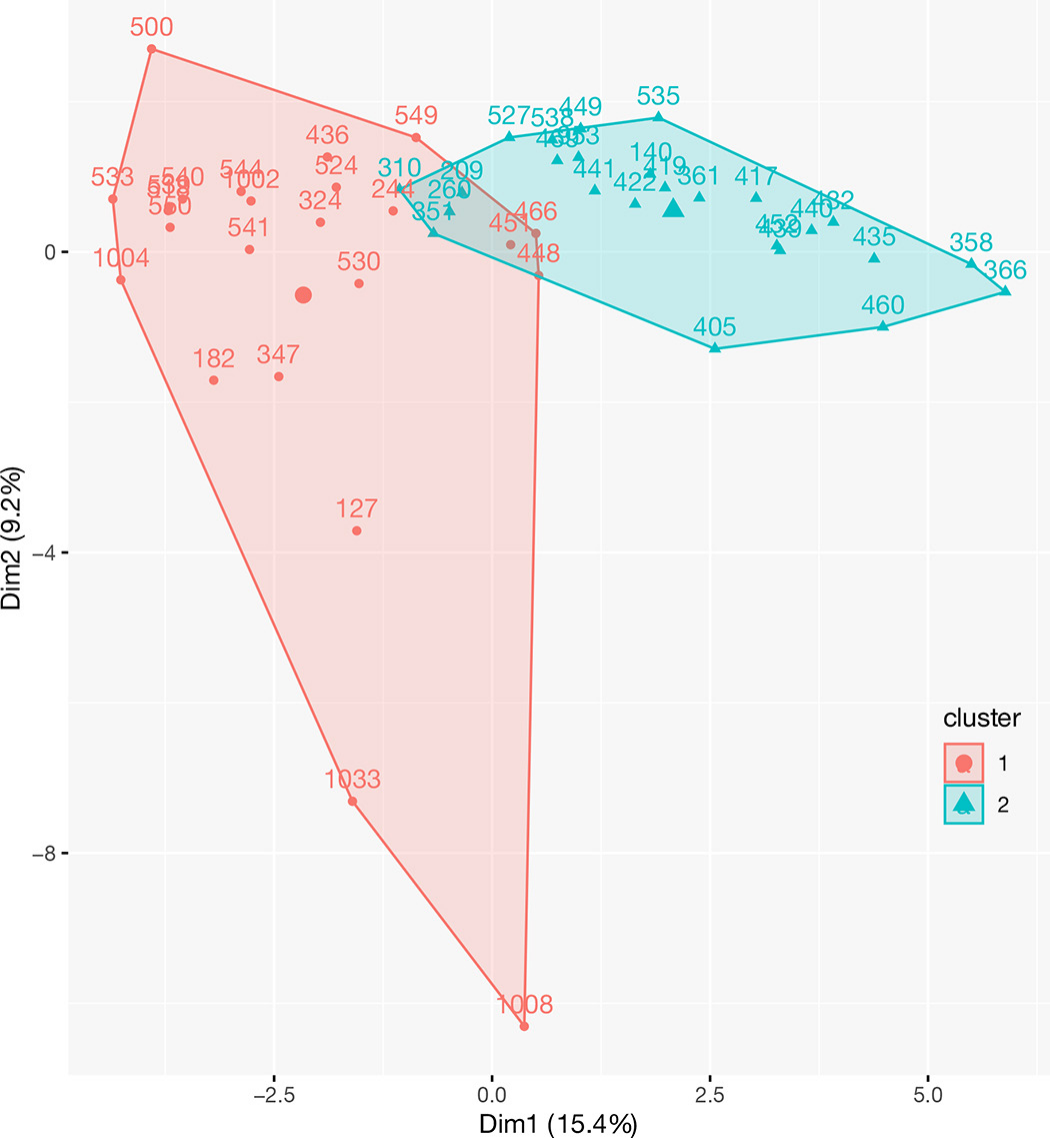
Principal component analysis plot depicting the clusters 1 (LR) and 2 (HR) using the data matrix.

Enteric gas emissions, feeding, production efficiency, and physiological traits were measured for LR and HR cows throughout the experimental period. Although the EME of both clusters were variable throughout the day (Figure 2), the daily average EME observed in HR cows (404 ± 6.04) was significantly lower (*P* = 0.003) than in LR cows (430 ± 6.27). The daily average CO_2_ emissions were significantly lower (*P* < 0.001) in LR cows (14,252 ± 202) than in HR cows (15,498 ± 194). Although H_2_ emissions were not significantly different, they were slightly lower in HR cows (3.54 ± 0.13) than in LR cows (3.84 ± 0.13). Moreover, to reflect production efficiency throughout the experimental period (Figure 3), EME (Figure 3A) were compared against DMI and MY to estimate the CH_4_ efficiency (Figure 3B) and CH_4_ intensity (Figure 3C), respectively. The CH_4_ efficiency (g CH_4_/kg DMI) and CH_4_ intensity (g CH_4_/kg milk) of LR cows (23.6 ± 0.64 and 13.9 ± 0.30) were both significantly different (*P* < 0.001) from HR cows (17.1 ± 0.64 and 10.3 ± 0.30). The DMI (kg/d) and MY (kg/d) for HR cows were 24.6 ± 0.28 and 40.6 ± 0.47, whereas for LR cows were 20.9 ± 0.28 and 35.1 ± 0.47, respectively. Regarding physiological responses, RT and ET exhibited significant differences (*P* < 0.001) between LR and HR cows throughout the day. The cows in the HR cluster ruminated 494 ± 31.6 min d^−1^ and ate 261 ± 15.9 min d^−1^, whereas the LR cluster ruminated 400 ± 24.4 min d^−1^ and ate 175 ± 10.7 min d^−1^.

**Figure 2:**
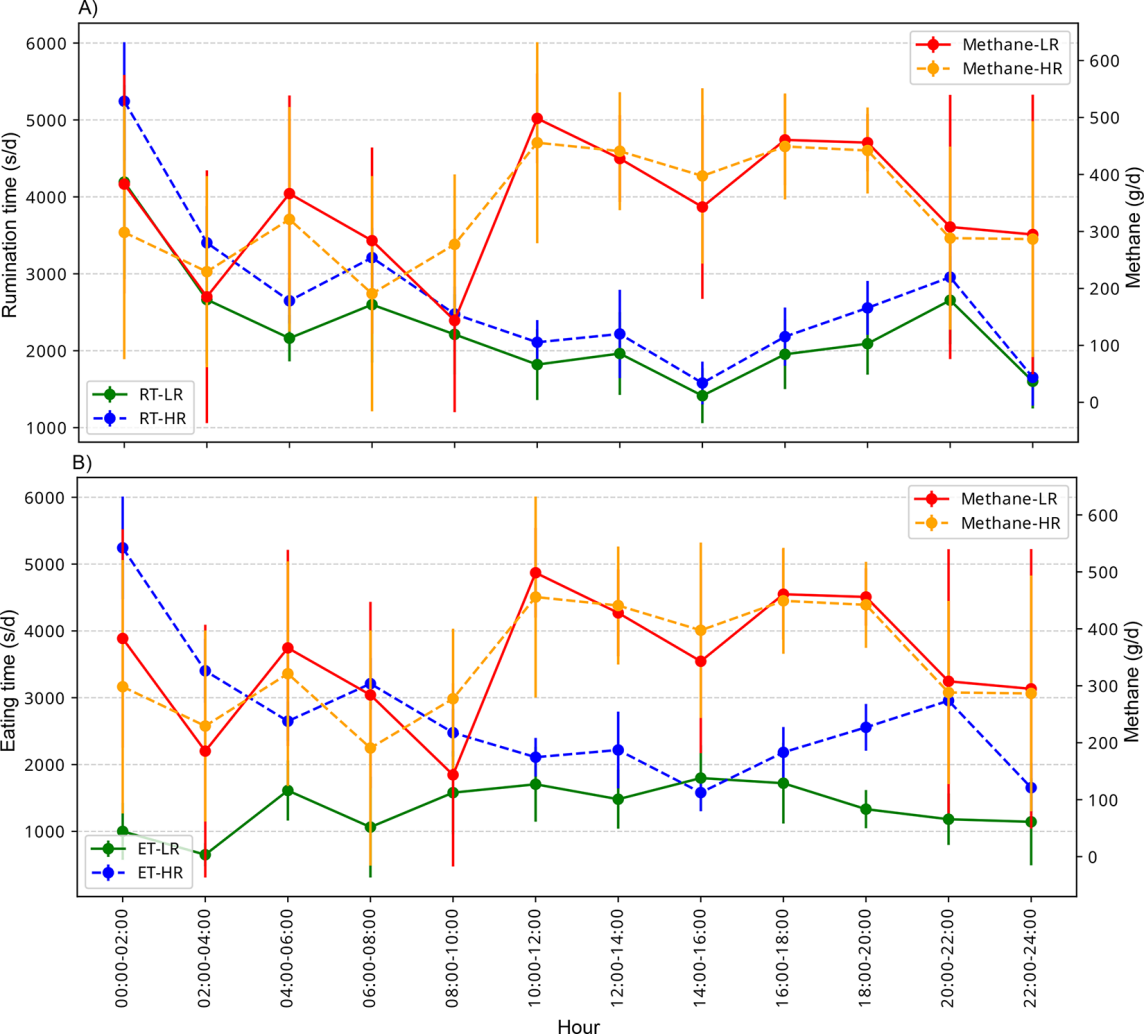
Circadian patterns shown every 2 h for RT (A) and ET (B) relative to CH_4_ yield of LR and HR cows during a 24-h cycle. The error bars indicate SD.

**Figure 3:**
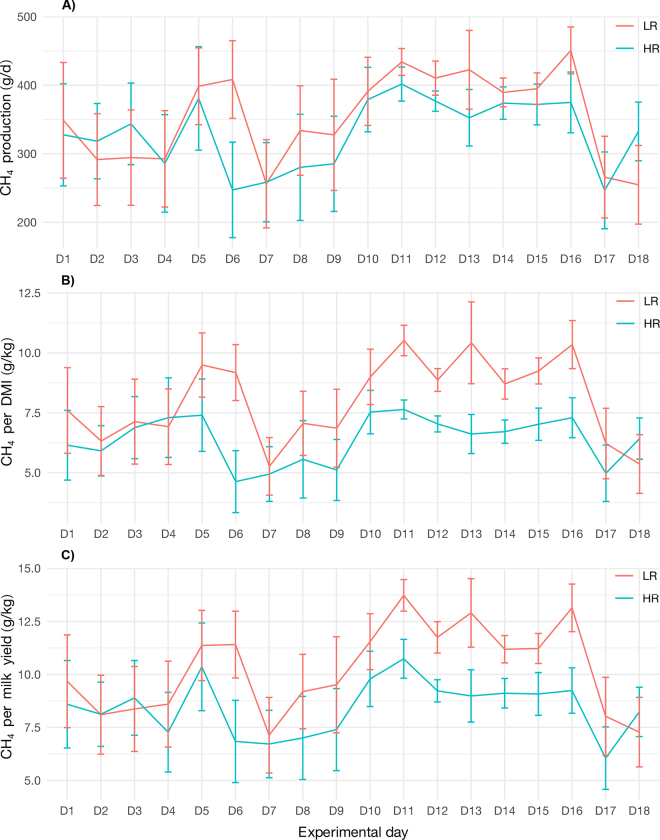
(A) CH_4_ production, (B) CH_4_ intensity, and (C) CH_4_ efficiency of LR and HR cows during the experimental period. The error bars represent SEM.

A correlation analysis was performed correlating RT and ET with EME; however, it did not provide meaningful results. Because we observed diurnal patterns on EME, RT, and ET, we repeated the analysis by time points. Across all experimental days, these data were categorized into 2-h intervals to determine whether there were specific time points where RT or ET were correlated with EME. Further, we also performed separate correlation analyses for LR and HR cows. The daily means of RT and ET were consistent throughout the experiment (data not shown). An inverse pattern between RT and ET was observed, meaning that rumination ceased when cows started eating and vice versa (Figure 2). Correlation between EME and RT (Figure 2) showed no strong association except for the 2 and 8 h time points postfeeding, although the 8 h time point showed a trend (*P* = 0.12) in LR cows. No strong correlations were found in HR cows. Overall, the cows in the HR cluster ruminated and ate more than those in the LR cluster. Although there were slight variations throughout the experiment between these clusters, the difference observed was consistent throughout the experiment.

We observed a diurnal pattern in EME with an increase soon after the morning feeding (1000 through 1200 h) and after the second milking (1600 through 1800 h). This pattern in EME was similar to the feeding pattern. These observations agree with the findings of Melgar et al. (2020). These authors reported a strong correlation between DMI and EME, which varies greatly throughout the day but was strongly correlated within 6 h postfeeding. Similarly, we observed that DMI and EME were correlated. Interestingly, Melgar et al. (2020) also reported a correlation between DMI and EME within the first 6 h in control cows, although no such correlations were noted under inhibited methanogenesis when cows were supplemented with 3-nitrooxypropanol (**3-NOP**). The LR cows are analogous to control cows, and 3-NOP cows are comparable to HR cows with the latter showing less EME and much lower EME per DMI compared with LR cows. It is worth noting that a trend (*P* < 0.12) was detected between EME and DMI in HR cows from 1600 to 1800 h.

Based on our novel method of classifying cows, we found that both clusters were significantly (*P* < 0.001) different for RT and ET and thus identified as HR and LR groups. The HR cows had higher RT, ruminating 94 min d^−1^ more than LR cows. Mikuła et al. (2022) also reported higher RT for HR cows than for LR cows, which is consistent with our findings. We found that HR cows had higher ET, eating 86 min d^−1^ more than LR cows. White et al. (2017) reported a mean ET of 284 min d^−1^, ranging between 141 and 507 min d^−1^, which is consistent with our study. We found that the MY of HR cows was higher, producing 5.5 kg d^−1^ more than LR cows. Likewise, we observed that EME between LR and HR cows were different. The EME, CH_4_ efficiency, and CH_4_ intensity of HR cows were all consistently lower throughout the experiment compared with LR cows. Our results are consistent with the studies of Mikuła et al. (2022), who reported that HR cows were more efficient than LR cows, emitting fewer grams of CH_4_ per day, per kilogram of DMI, and per kilogram of milk produced. Our findings showed that RT was higher in HR cows compared with LR cows and this may be supported by higher passage rates resulting in faster emptying rates and lower EME. These findings are supported by Goopy et al. (2014) and Hammond et al. (2014) who reported that sheep that had low EME also had a short retention time. Further, a report by Zhang et al. (2023) also showed that dairy cows with increased salivation had low EME and a small particle size. This chain of events also explains why in our study the MY of HR cows was higher than in LR cows, resulting in higher production efficiency and lower CH_4_ intensity due to fewer grams of CH_4_ emitted per kilogram of milk produced. Hence, further studies may consider measuring the passage rate between LR and HR cows.

This is the first study reporting the use of the DTW model to classify cows based on RT and ET. Clustering based on the cows' RT and ET patterns was repeatable, meaning that the DTW model identified cows differing in RT and ET statuses. Because the LR and HR groups were mostly composed of primiparous and multiparous cows, respectively, the RT and ET between groups were different, which was reflected by the distinct clustering patterns. Particularly, multiparous cows in the HR group clustered with primiparous cows in the LR group. Overall, there was a 90-min gap between the LR and HR clusters, repeatedly indicating that the DTW model is a suitable model for identifying LR and HR cows. Thus far, the only information available applying the DTW model in dairy cattle is on lame cows. The studies of Lide et al. (2020) and Apinan and Kuankid (2016) showed that the DTW model was precise, sensitive, and accurate for differentiating lame from sound cows based on the acceleration signals of the cows' hind legs and time series. This evidence indicates that although the DTW model has been used for other applications related to dairy cattle, this is the first study reporting the use of the DTW model to classify cows based on RT and ET. Thus, the DTW model is a tool to identify cows for RT and ET phenotypes.

It is known that cows spend more time eating during the day, whereas they ruminate more during the night (Paudyal et al., 2016). These physiological patterns have been reported by Heinrichs et al. (2021). This report shows that cows spend more time ruminating during the night with smaller rumination episodes during the day, confirming our findings. However, rumination and eating patterns can be influenced by several factors. Although the diet's feed composition has been reported to influence the cows' rumination and eating patterns (Salfer et al., 2018), factors inherent to the host can also influence them. Rumination is a moderately heritable trait that generates distinct rumination patterns among cows. Because rumination patterns are cyclical and nonlinear, artificial intelligence–based models such as the DTW model are appropriate for tracking and revealing these irregular patterns which are hidden in complex datasets. When the DTW model was applied to RT and ET datasets, we observed that the cows showed distinct rumination patterns despite being subjected to the same management and consuming the same diet. Moreover, the DTW model differentiated cows based on their rumination patterns, which were classified into the LR and HR clusters. This process was conducted on a sample of cows within the university's herd twice, indicating that the cows' rumination patterns remain constant throughout their lactation and that the clustering remains repeatable despite diet or physiological changes. Thus, the cows' rumination patterns can be considered as “fingerprints” to detect associations with other important phenotypes such as the CH_4_ yield phenotype.

Based on our findings, 3 major conclusions can be drawn. First, our results indicate that the DTW model is a suitable tool for classifying animals based on their RT and ET statuses relative to conventional statistical methods. Second, these physiological responses are reliable proxies for identifying animals with low CH_4_-emitting potential. Last, HR cows had consistently higher RT, ET, DMI, and MY, and lower EME and CH_4_ intensity than LR cows. Selecting cows with high RT concomitantly selects for low CH_4_ emitting potential, hence increasing the dairy herd's productivity and efficiency. This study provides an alternative method for classifying animals based on their RT and ET, offering the possibility of expanding the screening of large dairy herds in a time- and cost-efficient manner.
